# Prevalence of frailty, cognitive impairment, and sarcopenia in outpatients with cardiometabolic disease in a frailty clinic

**DOI:** 10.1186/s12877-018-0955-4

**Published:** 2018-11-06

**Authors:** Yoshiaki Tamura, Joji Ishikawa, Yoshinori Fujiwara, Masashi Tanaka, Nobuo Kanazawa, Yuko Chiba, Ai Iizuka, Sho Kaito, Jun Tanaka, Masamitsu Sugie, Takashi Nishimura, Akiko Kanemaru, Keigo Shimoji, Hirohiko Hirano, Ko Furuta, Akihiko Kitamura, Satoshi Seino, Shoji Shinkai, Kazumasa Harada, Shunei Kyo, Hideki Ito, Atsushi Araki

**Affiliations:** 1 Department of Diabetes, Metabolism, and Endocrinology, Tokyo Metropolitan Geriatric Hospital, Tokyo, Japan; 2 Department of Cardiology, Tokyo Metropolitan Geriatric Hospital, Tokyo, Japan; 3 Research Team for Social Participation and Community Health, Tokyo Metropolitan Institute of Gerontology, Tokyo, Japan; 4 Department of Clinical Laboratory, Tokyo Metropolitan Geriatric Hospital, Tokyo, Japan; 5 Department of Surgery, Tokyo Metropolitan Geriatric Hospital, Tokyo, Japan; 6 Department of Cardiac Surgery, Tokyo Metropolitan Geriatric Hospital, Tokyo, Japan; 7 Department of Rehabilitation, Tokyo Metropolitan Geriatric Hospital, Tokyo, Japan; 8 Department of Diagnostic Radiology, Tokyo Metropolitan Geriatric Hospital, Tokyo, Japan; 9 Department of Dentistry and Oral Surgery, Tokyo Metropolitan Geriatric Hospital, Tokyo, Japan; 10 Department of Psychiatry, Tokyo Metropolitan Geriatric Hospital, Tokyo, Japan

**Keywords:** Frailty, Frailty clinic, Modified CHS criteria, Cognitive impairment, Sarcopenia, Cardiometabolic diseases

## Abstract

**Background:**

Although frailty and cognitive impairment are critical risk factors for disability and mortality in the general population of older inhabitants, the prevalence and incidence of these factors in individuals treated in the specialty outpatient clinics are unknown.

**Methods:**

We recently established a frailty clinic for comprehensive assessments of conditions such as frailty, sarcopenia, and cognition, and planned 3-year prospective observational study to identify the risk factors for progression of these aging-related statuses. To date, we recruited 323 patients who revealed symptoms suggestive of frailty mainly from a specialty outpatient clinic of cardiology and diabetes. Frailty status was diagnosed by the modified Cardiovascular Health Study (mCHS) criteria and some other scales. Cognitive function was assessed by Mini-Mental State Examination (MMSE), Japanese version of the Montreal Cognitive Assessment (MoCA-J), and some other modalities. Sarcopenia was defined by the criteria of the Asian Working Group for Sarcopenia (AWGS). In this report, we outlined our frailty clinic and analyzed the background characteristics of the subjects.

**Results:**

Most patients reported hypertension (78%), diabetes mellitus (57%), or dyslipidemia (63%), and cardiovascular disease and probable heart failure also had a higher prevalence. The prevalence of frailty diagnosed according to the mCHS criteria, cognitive impairment defined by MMSE (≤27) and MoCA-J (≤25), and of AWGS-defined sarcopenia were 24, 41, and 84, and 31%, respectively. The prevalence of frailty and cognitive impairment increased with aging, whereas the increase in sarcopenia prevalence plateaued after the age of 80 years. No significant differences were observed in the prevalence of frailty, cognitive impairment, and sarcopenia between the groups with and without diabetes mellitus, hypertension, or dyslipidemia with a few exceptions, presumably due to the high-risk subjects who had multiple cardiovascular comorbidities. A majority of the frail and sarcopenic patients revealed cognitive impairment, whereas the frequency of suspected dementia among these patients were both approximately 20%.

**Conclusions:**

We found a high prevalence of frailty, cognitive impairment, and sarcopenia in patients with cardiometabolic disease in our frailty clinic. Comprehensive assessment of the high-risk patients could be useful to identify the risk factors for progression of frailty and cognitive decline.

## Background

Recently, although life expectancies in the developed countries, including Japan, have been increasing, the number of older people with functional disabilities who need assistance from others is also on rise. Extending healthy life expectancy is an urgent task for the gerontologists.

Frailty is a state in which an older person becomes vulnerable to the external stresses due to declining age-related physiological reserve and can lead to disabilities, falls, fractures, and death [1, 2]. Frailty is a reversible condition because physical and nutritional intervention can improve a person’s physical condition. The concept of multidimensional frailty based on a comprehensive geriatric assessment has been proposed because cognitive and social frailties, as well as physical frailty, have a major effect on disability and mortality. Thus, it is essential to screen for frailty and cognitive deficits in the older people to prevent deterioration of their functional ability.

The prevalence of frailty has been reported to be approximately10% in the general population of older inhabitants. Although cardiometabolic diseases [diabetes mellitus (DM), hypertension (HT), dyslipidemia (DL), and heart failure] have been associated with the prevalence of frailty in epidemiological studies, this prevalence in the individuals treated in the cardiology and diabetes specialty outpatient clinics remains unknown.

However, it is difficult to complete the multidimensional assessment of frailty during the routine visits in the outpatient clinics. Therefore, we recently established a frailty clinic and identified a cohort group of patients in the clinic for inclusion in a 3-year prospective longitudinal study.

The aim of this prospective study was to answer the following questions: first, what is the prevalence and incidence of frailty in the specialized frailty clinic? what are the associations, if any, between frailty status and clinical outcomes of fall, cardiovascular disease, dementia, hospitalization, functional disability, and death? and what are the most useful indices for predicting these outcomes in evaluating frailty status?

In this article, we describe our frailty clinic and the baseline characteristics of the patients in a cohort for the prospective longitudinal study.

## Methods

### Frailty clinic

Our frailty clinic was opened to comprehensively assess frailty, sarcopenia, cognition, psychological condition, nutrition, medications, and social status of patients in October 2015. At present, doctors from the departments of endocrinology and cardiology examine the patients in the frailty clinic in a rotational system. One to two clinical psychologists are present in the clinic every day to interview the patients.

### Subjects

**Fig. 1:**
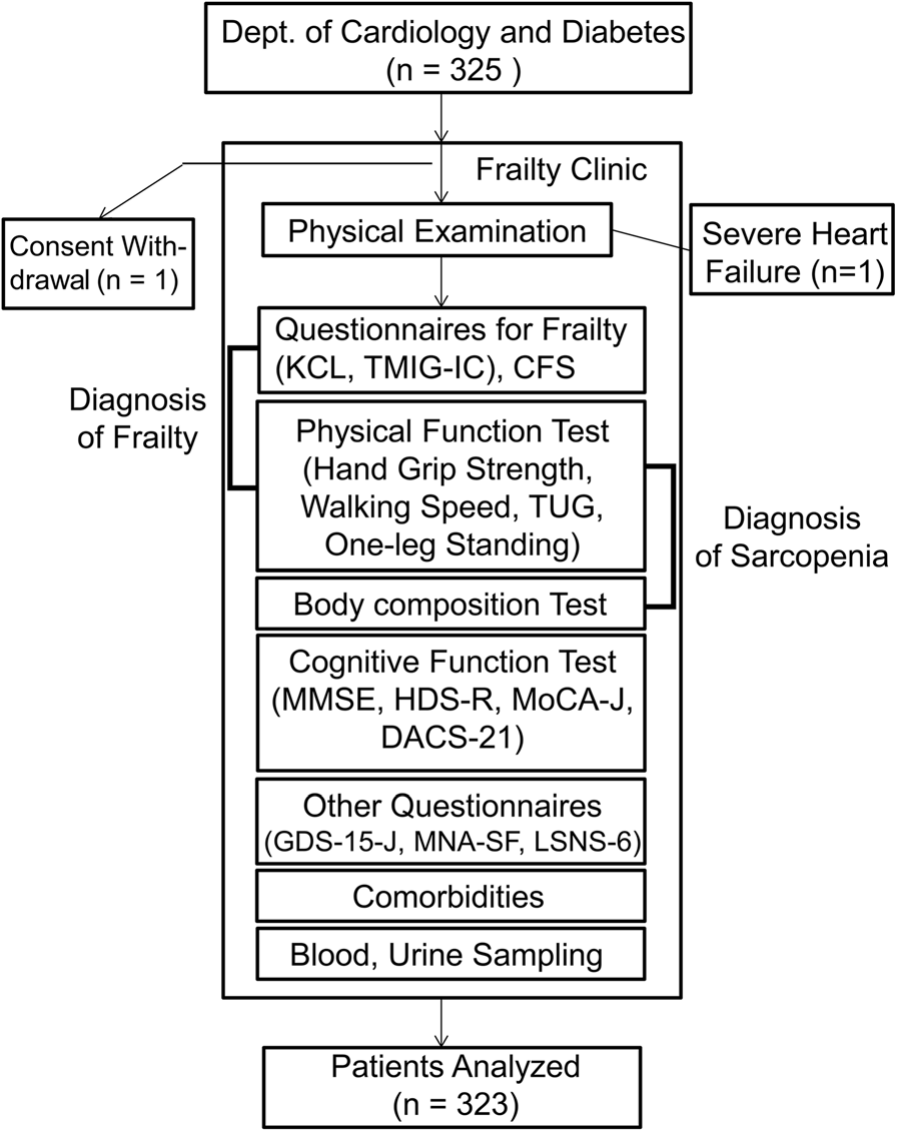
Flow chart of the recruitment of participants and assessment methods. KCL, Kihon Check List; TMIG-IC, Tokyo Metropolitan Institute of Gerontology Index of Competence; CFS, Clinical Frailty Scale; TUG, Timed Up and Go Test; MMSE, Mini-Mental State Examination; HDS-R, Revised Hasegawa’s Dementia Scale; MoCA-J, Japanese version of the Montreal Cognitive Assessment; DASC-21, Dementia Assessment Sheet in Community-based Integrated Care System-21 items; GDS-15-J, Japanese version of the Geriatric Depression Scale 15; MNA-SF, Mini-Nutritional Assessment-Short Form; LSNS-6, Lubben Social Network Scale-6

Three hundred twenty-five patients were recruited mainly from the outpatient clinics of cardiology and diabetes departments of our hospital who gave their consent to be assessed for frailty. When the subjects initially visited the frailty clinic, informed consent was obtained for inclusion in a planned 3-year observational study. After a short interview to gather information on their medical history, family history, and life history, a brief systematic physical examination was performed by a physician to identify any underlying disease. Patients who revealed a history of advanced cancer, acute severe diseases or conditions requiring hospitalization, and severely impaired activities of daily living (ADL) and/or cognitive function, were excluded. Only one patient was excluded by these criteria, because of severe heart failure. Patients who were free of these diseases were subjected to questionnaires, physical function tests, and a body composition assessment, as described below. All patients underwent the same assessments. The flow chart of the method is shown in Fig. 1. Six hospitalized patients with DM and HT, who were generally stable, were also included. One subject withdrew the consent. Finally, a total of 323 patients were enrolled for the analysis in this study.

In this report, the subjects were registered as the first cohort between June 22, 2015 and Mar 10, 2017; however, as this is an ongoing study, the number of subjects will increase to up to 800 as the final cohort.

### Frailty status

Frailty status was evaluated according to the following four criteria: I) The modified version of the Cardiovascular Health Study (CHS) criteria (mCHS). CHS criteria were originally proposed by Fried et al. [1] and comprised five indices of frailty: weight loss, exhaustion, weakness, inactivity, and low walking speed. We modified the CHS based on the report by Makizono et al. [3] in which their criteria were adapted to a Japanese population. For assessing weakness, hand grip strength < 26 kg (males) and < 18 kg (females) were set as the cutoff points regardless of the body mass index (BMI). To evaluate the slow gait speed, 4-m walk tests were administered, and a walk speed < 1.0 m/s, regardless of sex or body height, was set as a cutoff. We further modified the CHS criteria by using certain questions in the Kihon Check List (KCL) [4]. For evaluating the body weight loss and exhaustion, the KCL questions, “Did you experience >2–3 kg of body weight loss in the last 6 months?” and “In the last 2 weeks, have you felt tired without a reason?” were asked. For the question of low physical activity, those who answered either “No” for the question “Do you go out at least once a week?” or “Yes” for the question “Do you go out less frequently than you did last year?” were defined as positive. Subjects who were positive in three of the five indices were diagnosed as frail, and those positive in one or two indices were diagnosed as prefrail.

II) Clinical Frailty Scale (CFS); patients were classified into nine categories based on their dependence on others [5]. We defined subjects whose CFS scores were ≥ 4 as frail, although in the original report, the term “frail” was used for scores ≥5.

III) KCL; the KCL was created by the Ministry of Health, Labor, and Welfare of the Japanese government for screening the older frail group and comprised 25 items that evaluate not only ADL and physical function but also nutrition, oral health, social withdrawal, cognition, and depression. Subjects whose scores were ≥ 8 were diagnosed as frail [4]. IV) The Tokyo Metropolitan Institute of Gerontology Index of Competence (TMIG-IC); the TMIG-IC was originally created by our institute to evaluate the higher-level functional capacity [6]. It comprises 13 items, evaluating the instrumental ADL (5 items), intellectual activity (4 items), and social role (4 items). Subjects whose scores were ≤ 9 were diagnosed as frail.

### Cognitive function assessment

The Mini-Mental State Examination (MMSE) and Hasegawa’s Dementia Scale-Revised (HDS-R) were used for the functional assessments. We also performed the Japanese version of the Montreal Cognitive Assessment (MoCA-J) [7]. We further performed the Dementia Assessment Sheet in Community-based Integrated Care System-21 items (DASC-21), a questionnaire set used to easily evaluate the impaired cognitive function and basic ADL at the same time [8]. Cognitive impairment was defined as an MMSE score ≤ 27 or an MoCA-J ≤ 25 [7, 9], and suspected dementia was defined as an MMSE score ≤ 23, HDS-*R* ≤ 20, or DASC-21 ≥ 31.

### Depressive mood, nutritional status, and social support network

The Japanese version of the Geriatric Depression Scale 15 (GDS-15-J) was used to evaluate depressive mood [10]. Subjects whose scores were ≥ 5 were suspected to have a depressive tendency. To evaluate the subjects’ nutritional status, the Mini-Nutritional Assessment-Short Form (MNA-SF) was used [11], which comprised 6 items, with a highest score of 14; subjects with scores from 8 to 11 were suspected to be at risk of malnutrition, whereas subjects with scores of ≤7 were suspected as being malnourished. The status of the subjects’ social support was evaluated by using the Japanese version of the Lubben Social Network Scale-6 (LSNS-6) [12]. Scores ranged from 0 to 30, and lower scores indicate a lack of social support.

### Physical performance tests

Hand grip, usual walking speed, timed up and go test (TUG), and one-leg standing were performed. Hand grip strength was measured by using a dynamometer (Takei Scientific Instruments Co., Ltd., Niigata, Japan) for both hands, and the best results were recorded. To measure usual walking speed, patients were instructed to walk for 6 m at their ordinary speed. The time spent for walking the middle 4 m was measured, and the walking speed was calculated. Hand grip and walking speed were measured twice, and the best result was recorded. Details of TUG have been described elsewhere [13]. Briefly, the time spent for the following series of movement was measured: standing up, walking (at a maximum speed) to a mark 3 m ahead, turning, walking (at a maximum speed) back to a seat, and sitting down. For one-leg standing, patients were instructed to stand on either of their legs for maximum duration, and the time was recorded. The TUG and one-leg standing test were administered twice, and the average value was recorded; however, for the one-leg standing test, if the better value was more than twice the other, the better value was recorded, and if a subject could stand for > 60 s for either of the two trials, the time was recorded as 60 s. If a subject could not complete the 4-m walking test or TUG, their data were omitted, whereas the time of those who could not stand on either leg was recorded as 0 s.

### Body composition test and diagnosis of sarcopenia

Body composition was evaluated by bioimpedance analysis using an InBody 770® (InBody Japan Inc., Tokyo, Japan). Skeletal muscle mass index (SMI) was calculated by dividing the appendicular muscle mass (kg) by the square of body height (m). Sarcopenia was diagnosed according to the criteria of the Asian Working Group for Sarcopenia (AWGS) [14]. Those who also exhibited low SMI in conjunction with either low grip strength or slow walking speed were diagnosed as having sarcopenia.

### Comorbidities

The concurrent diseases were diagnosed by descriptions in the clinical records. History of coronary artery disease (CAD) was defined as a history of either angina pectoris or myocardial infarction or both. History of stroke was defined as a history of either cerebral infarction or cerebral bleeding or both. Probable heart failure was diagnosed either by the clinical record or as brain natriuretic peptide (BNP) values ≥100 pg/mL.

### Blood sampling

Blood was collected ad libitum. Blood cell count, blood biochemistry tests, and measurement of glycohemoglobin (HbA1c) and plasma BNP were performed as normally done in the clinic. Serum was preserved at − 20 °C for further investigation.

### Other evaluation tests

Self-measured blood pressure at home, ambulatory blood pressure monitoring, central arterial pressure, ankle brachial pressure index, pulse wave velocity, carotid Doppler ultrasonography, echocardiography, brain magnetic resonance imaging, lower extremity motor function analysis to measure power, speed, and balance during a standing-up motion (zaRitz®; Tanita Corp., Tokyo, Japan) and an autonomic nervous function test (Kiritsu-Meijin®; Crosswell Co., Ltd., Kanagawa, Japan) were performed in some patients when necessary. To assess the quality of life of health status, the Japanese version of the questionnaire EQ-5D-5 L was used [15]. To evaluate physical activity, the International Physical Activity Questionnaire was used [16]. To assess the frequency of going outdoors and social participation, questionnaires adopted in the previous reports were used [17, 18]. In addition, the subjects were asked if they had a certified level of support or care needs or if they had received any long-term care services from the health insurance system in Japan.

### Outcomes of the longitudinal study

During the 3-year longitudinal observational study, the following outcomes were evaluated annually using questionnaires and medical charts: (1) incidence of fall and fracture; (2) incidence or progression of frailty status; (3) dementia; (4) cardiovascular disease (myocardial infarction, stroke, cardiovascular interventions); (5) hospitalization; (6) certified level of support or long-term care needs from the insurance system; and (7) death.

### Statistical analysis

To test the difference in the frequencies between groups of categorical data, we used the chi-square test. To compare the continuous valuables between the two groups, we used the Mann–Whitney test. All statistical analyses were performed by using the SPSS Statistics 20 software package (IBM, Armonk, NY, USA). In all comparisons, the significance level was set at *P* < 0.05.

## Results

### Background of the subjects

The study participants included 323 patients who visited the frailty clinic. The characteristics of the subjects are summarized in Table 1. These patients were aged between 50 and 95 years, but 97% were ≥ 65 years, with a median age of 78 years. Reflecting the background of the subjects recruited from the endocrinology and cardiology departments, the major comorbidities of the subjects were the metabolic and cardiac diseases, including HT (78%), DM (57%), dyslipidemia (DL, 63%), CAD (18%), stroke (11%), and probable heart failure (22%). Scores of GDS-15-J, MNA-SF, and the Japanese version of LSNS-6 are also summarized in Table 1. Forty-eight percent of the patients scored ≥5 points in the GDS-15-J. Nutritional statuses were fairly good in this population.

**Table 1:** Background characteristics of subjects (*n* = 323)

Age (y)	78 (75–82)
Male (%)	37.8
BMI (kg/m^2^)	23.0 (21.3–25.5)
Systolic BP (mmHg) (*n* = 320)	130 (120–140)
Diastolic BP (mmHg) (*n* = 320)	74 (65–82)
HbA1c (%) (*n* = 321)	6.4 (5.9–7.1)
TC (mg/dl) (*n* = 321)	191 (168–215)
TG (mg/dl) (*n* = 321)	119 (84–164)
HDL-cholesterol (mg/dl) (*n* = 321)	55 (47–66)
eGFR (mL/min/1.73m^2^) (*n* = 321)	58 (47–68)
BNP (pg/mL) (*n* = 287)	35 (21–70)
Hypertension (%) (*n* = 320)	78.0
Diabetes Mellitus (%)	57.3
Dyslipidemia (%)	62.5
CAD (%)	18.0
Stroke (%) (*n* = 312)	10.9
Probable Heart Failure (%) (*n* = 288)	22.2
GDS-15-J (*n* = 322)	4 (2–7)
MNA-SF (*n* = 322)	12 (10–13)
LSNS-6 (*n* = 309)	12 (8–16)

*Abbreviations*: *BMI* body mass index*, BP* blood pressure*, HbA1c* glycohemoglobin*, TC* total cholesterol*, TG* triglyceride*, HDL* high-density lipoprotein*, eGFR* estimated glomerular filtration rate*, CAD* coronary artery disease*, GDS-15-J* Japanese version of the Geriatric Depression Scale 15*, MNA-SF* Mini-Nutritional Assessment-Short Form*, LSNS-6* Lubben Social Network Scale-6

For continuous variables, values indicate median (25–75th percentile)

### Prevalence of frailty, cognitive impairment, and sarcopenia

The prevalence of frailty and cognitive impairment is summarized in Table 2. The prevalence of subjects who were robust, prefrail, and frail diagnosed by the mCHS were 26, 50, and 24%, respectively. According to the CFS, KCL, and TMIG-IC criteria, 32, 34, and 27%, respectively, of the subjects were diagnosed as being frail.

**Table 2:** Background characteristics of frailty and cognitive function

Frailty status	
mCHS status (*n* = 303)	
Robust (%)	26.1
Prefrailty (%)	49.8
Frailty (%)	24.1
Frailty (%) by CFS (*n* = 315)	31.7
Frailty (%) by KCL (*n* = 311)	33.8
Frailty (%) by TMIG-IC (*n* = 320)	26.6
Cognitive function	
MMSE (*n* = 320)	28 (26–29)
HDS-R (*n* = 320)	27 (24–29)
MoCA-J (*n* = 320)	22 (19–25)
DASC-21 (*n* = 264)	24 (23–27)
* Cognitive impairment*	
MMSE ≤27 (%)(*n* = 320)	40.9
MoCA -J ≤ 25 (%)(*n* = 320)	84.1
* Suspected dementia*	
MMSE ≤23 (%)(*n* = 320)	12.8
HDS-R ≤ 20 (%)(*n* = 320)	11.6
DASC-21 ≥ 31 (%)(*n* = 264)	14.8

*Abbreviations*: *SMI* Skeletal Muscle Mass Index, *TUG* Timed up and go test For continuous variables, values indicate median (25–75^th^ percentile)

The median MMSE, HDS-R, MoCA-J, and DASC-21 scores were 28, 27, 22, and 24, respectively. The prevalence of cognitive impairment was higher than that for suspected dementia but was significantly different between the evaluation methods; the assessment by an MMSE score ≤ 27 revealed prevalence of 41%, while the prevalence according to the criteria of MoCA-J ≤ 25 was 84%. The prevalence of suspected dementia was comparable between the evaluation methods; assessment by an MMSE score ≤ 23, HDS-*R* ≤ 20, and DASC-21 ≥ 31 revealed prevalence of 13, 12, and 15%, respectively.

The results of body composition, physical performance tests and sarcopenia are summarized in Table 3. The prevalence of sarcopenia in males and females were 33 and 30%, respectively. For the diagnostic elements, approximately half of the patients matched the criteria of low SMI and low hand grip in both sexes. In contrast, the number of patients with low gait speed was significantly small.

**Table 3:** Background characteristics of body composition, physical function, and sarcopenia

	Total	Male	Female
SMI (kg/m^2^)	6.3 (5.6–7.0) (*n* = 312)	7.0 (6.6–7.7) (*n* = 119)	5.8 (5.3–6.4) (*n* = 193)
Hand Grip (kg)	20.3 (16.1–25.5) (*n* = 313)	27.0 (22.3–32.3) (*n* = 118)	17.5 (14.5–20.9) (*n* = 195)
Walk Speed (m/s)	1.11 (0.91–1.29) (*n* = 312)	1.11 (0.90–1.28) (*n* = 119)	1.11 (0.91–1.30) (*n* = 193)
Low SMI (%)	45.2 (*n* = 312)	48.7 (*n* = 119)	43.0 (*n* = 193)
Low Hand Grip (%)	49.5 (*n* = 313)	44.9 (*n* = 118)	52.3 (*n* = 195)
Slow Walk Speed (%)	15.4 (*n* = 312)	13.4 (*n* = 119)	16.6 (*n* = 193)
Sarcopenia (%)	31.4 (*n* = 309)	33.1 (*n* = 118)	30.4 (*n* = 191)
TUG (s)	7.7 (6.6–9.6) (*n* = 301)	7.3 (6.2–9.3) (*n* = 116)	7.9 (6.7–9.8) (*n* = 185)
One Leg Standing (s)	7.1 (2.3–24.4) (*n* = 312)	6.9 (2.5–25.2) (*n* = 119)	7.1 (2.1–23.6) (*n* = 193)

*Abbreviations*: *mCHS* modified Cardiovascular Health Study, *CFS* Clinical Frailty Scale, *KCL* Kihon Check List, *TMIG-IC* Tokyo Metropolitan Institute of Gerontology Index of Competence, *MMSE* Mini-Mental State Examination, *HDS-R* Hasegawa’s Dementia Scale for Revised, *MoCA-J* Japanese version of the Montreal Cognitive Assessment, *DASC-21* Dementia Assessment Sheet in Community-based Integrated Care System-21 itemsFor continuous variables, values indicate median (25–75^th^ percentile)

### Prevalence of frailty, cognitive impairment, and sarcopenia stratified by age

**Fig. 2:**
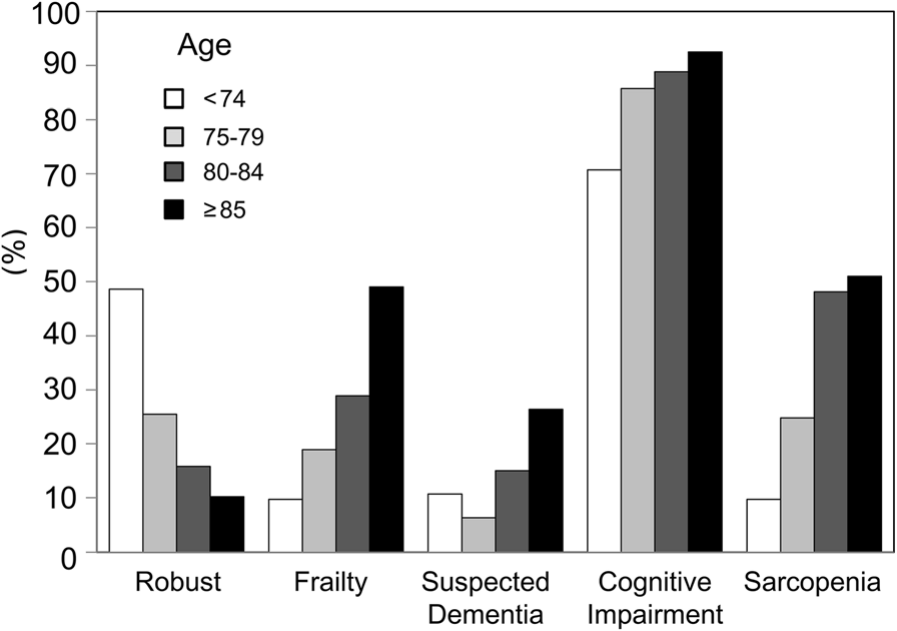
Frequencies of frailty, suspected dementia (MMSE score ≤ 23), cognitive impairment (MoCA-J ≤ 25), and sarcopenia stratified by age

Figure 2 presents the prevalence of frailty, suspected dementia, cognitive impairment, and sarcopenia stratified by age categories of 65–74, 75–79, 80–84, and ≥ 85 years. The percentage of robust subjects diagnosed according to the mCHS criteria decreased significantly with the increasing age, whereas the percentages of subjects who were frail and had suspected dementia, cognitive impairment, and sarcopenia, were all significantly increased with the increasing age. Almost half of the subjects who were ≥ 85 years old were frail, and the prevalence of cognitive impairment (defined by the MoCA-J ≤ 25) and sarcopenia in subjects ≥80 years were approximately 90 and 50%, respectively. The increase in sarcopenia prevalence plateaued after the age of 80 years.

### Prevalence of patients with frailty, sarcopenia, and cognitive impairment among the DM, HT, and DL subjects

Tables 4 and 5 summarizes the prevalence of patients with frailty (Table 4), suspected dementia, cognitive impairment and sarcopenia (Table 5) compared between those with and without DM, with and without HT, and with and without DL stratified by age*.* There were no significant differences, except for the significantly higher and lower prevalence of sarcopenia in the ≤74 years age group of DM and the 80–84 years age group of HT, respectively.

**Table 4:** Frequency of frailty diagnosed by mCHS criteria among DM, HT, and DL patients [values indicate number (%)]

Age	Frailty Status	DM (−)	DM (+)	*P* value	HT (−)	HT (+)	P value	DL (−)	DL (+)	*P* value
50–74	Robust	18 (60)	17 (41)	0.147	10 (50)	25 (48)	0.577	13 (59)	22 (44)	0.480
	Prerail	11 (37)	19 (45)		7 (35)	23 (44)		7 (32)	23 (46)	
	frail	1 (3)	6 (14)		3 (15)	4 (8)		2 (9)	5 (10)	
75–79	Robust	12 (30)	15 (23)	0.706	5 (26)	22 (25)	0.950	7 (20)	20 (28)	0.381
	Prerail	21 (53)	38 (58)		10 (53)	49 (56)		19 (54)	40 (56)	
	frail	7 (18)	13 (20)		4 (21)	16 (18)		9 (26)	11 (16)	
80–84	Robust	4 (11)	8 (20)	0.327	1 (6)	11 (19)	0.393	5 (17)	7 (15)	0.740
	Prerail	19 (53)	23 (58)		11 (61)	31 (53)		15 (50)	27 (59)	
	frail	13 (36)	9 (23)		6 (33)	16 (27)		10 (33)	12 (26)	
≥85	Robust	4 (14)	1 (5)	0.113	2 (17)	3 (8)	0.412	2 (7)	3 (14)	0.755
	Prerail	8 (29)	12 (57)		6 (50)	14 (38)		11 (41)	9 (41)	
	frail	16 (57)	8 (38)		4 (33)	20 (54)		14 (52)	10 (55)	

*Abbreviations*: *DM* diabetes mellitus*, HT* hypertension*, DL* dyslipidemia

**Table 5:** Frequency of suspected dementia, cognitive impairment, and sarcopenia among DM, HT, and DL patients [values indicate number (%)]

Age	DM (-)	DM (+)	*P* value	HT (-)	HT (+)	*P* value	DL (-)	DL (+)	*P* value
Frequency of suspected dementia									
50–74	3 (10)	5 (11)	1.000	2 (10)	6 (11)	1.000	4 (17)	4 (8)	0.259
75–79	4 (10)	3 (4)	0.257	2 (10)	5 (5)	0.606	0 (0)	7 (9)	0.094
80–84	5 (14)	7 (16)	1.000	3 (16)	9 (15)	1.000	5 (16)	7 (14)	1.000
≥85	8 (27)	6 (26)	1.000	2 (15)	12 (30)	0.473	8 (28)	6 (25)	1.000
Frequency of cognitive impairment									
50–74	19 (61)	34 (77)	0.198	13 (65)	40 (73)	0.572	17 (71)	36 (71)	1.000
75–79	34 (83)	62 (87)	0.580	15 (75)	81 (88)	0.159	31 (86)	65 (86)	1.000
80–84	31 (86)	40 (91)	0.724	16 (84)	55 (90)	0.437	29 (94)	42 (86)	0.470
≥85	29 (97)	20 (87)	0.305	11 (85)	38 (95)	0.249	28 (97)	21 (88)	0.318
Frequency of sarcopenia									
50–74	0 (0)	7 (17)	0.017*	3 (15)	4 (8)	0.388	2 (9)	5 (10)	1.000
75–79	7 (18)	20 (29)	0.250	6 (32)	21 (23)	0.559	11 (32)	16 (21)	0.238
80–84	17 (50)	20 (47)	0.821	14 (78)	23 (39)	0.006**	13 (45)	24 (50)	0.814
≥85	16 (53)	10 (48)	0.779	7 (58)	19 (49)	0.743	16 (55)	10 (46)	0.577

*Abbreviations*: *DM* diabetes mellitus*, HT* hypertension*, DL* dyslipidemia

*p < 0.05. **p < 0.01

### Overlap of frailty, sarcopenia, and cognitive impairment/suspected dementia

**Fig. 3:**
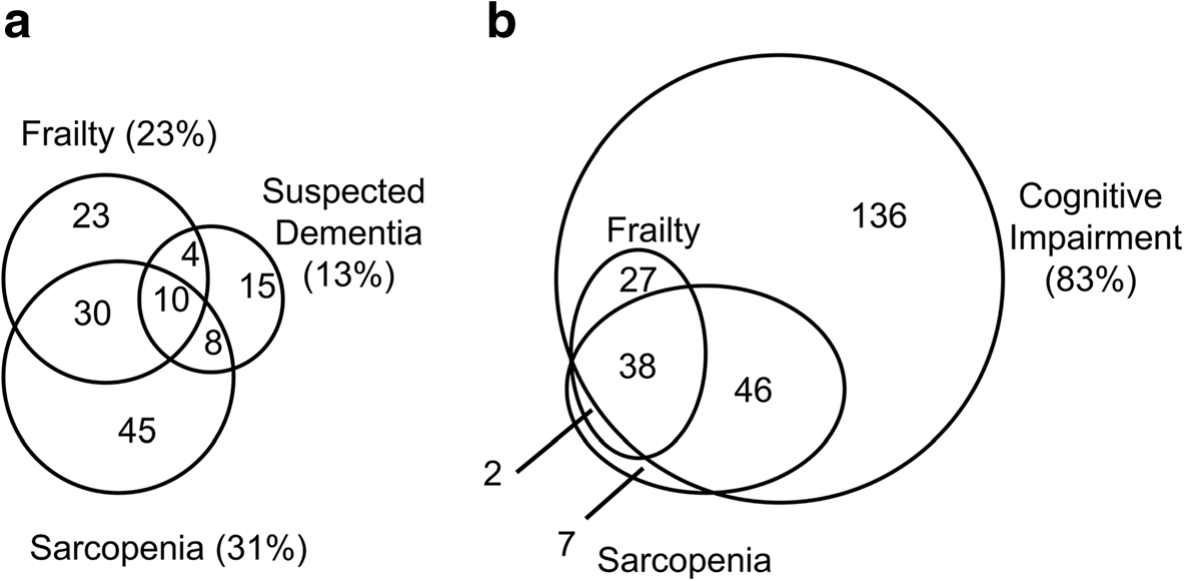
Overlap of frailty, sarcopenia, and suspected dementia (MMSE score ≤ 23) (**a**) and overlap of frailty, sarcopenia, and cognitive impairment (MoCA-J ≤ 25) (**b**). Numbers indicates the number of patients included in the area

The overlap of frailty, sarcopenia, and suspected dementia (MMSE ≤23)/cognitive impairment (MoCA-J ≤ 25) are presented in Fig. 3a and b. Approximately, 60% of the frail subjects were also sarcopenic and 40% of those with sarcopenia were also frail. Of interest, patterns of the diagrams are significantly different in relation to the cognitive function. Approximately, 20% each of the subjects with frailty or with sarcopenia were also diagnosed as having suspected dementia, whereas almost all of the frail (97%) and sarcopenic (90%) subjects were diagnosed as having cognitive impairment.

## Discussion

In this study, we described our recently established frailty clinic, which mainly treats patients with cardiometabolic diseases. At present, there is an urgent need to assess frailty and cognition in older individuals with cardiometabolic disease because diabetes and cardiovascular disease are associated with the aging process and with frailty and cognitive impairment [19]. We performed a comprehensive geriatric assessment for these patients to evaluate frailty, suspected dementia, cognitive impairment, and sarcopenia and found that the prevalence of all of these conditions increased with increasing age (Fig. 2).

The prevalence of frailty diagnosed according to the mCHS criteria in our study population was about twice as high as that of the recently reported community-dwelling older persons, and in most of those, the prevalence was approximately 10% [20–22]. One reason for the discrepancy was the difference in the diagnostic criteria. We modified the cutoff points for SMI, grip strength, and walking speed, and we substituted some questions from the KCL for the original ones since they were easier to obtain answers from the subjects; however, the major reasons for the discrepancy was that our study populations included higher rate of patients aged over 80 and that they comprised outpatients, especially those who had cardiometabolic diseases, since most of them were recruited from the departments of cardiology or diabetes.

In this report, we attempted to evaluate frailty status using several different scales, but the prevalence of frailty varied from 24 to 34% depending on the scale. The highest prevalence was observed in the KCL criteria, which was 10% higher than that in the mCHS criteria, perhaps because the KCL questionnaire assesses multidimensional aspects of frailty, including physical, cognitive, and social frailty; malnutrition; poor oral health; and depression. The high prevalence of cardiometabolic diseases could explain this discrepancy, particularly with DM, which is frequently associated with physical frailty as well as mental disorders, as is the case with the TMIG-IC criteria, which includes questionnaires regarding intellectual activity and social roles. The CFS criterion showed a high prevalence of frailty when we defined it using a cutoff value of ≥4 instead of the original cutoff value of > 5 (the prevalence rate was reduced to 8.6% when we diagnosed frailty using the ≥5 cutoff). In a prospective study, we believe that all of these results will be useful in clarifying the most appropriate diagnostic criteria for predicting functional disability or mortality in patients with cardiovascular risk factors.

Although a few patients were suspected of having dementia, there were a substantial number of patients with mildly impaired cognitive function, and the frequency of the MoCA-J ≤ 25 was revealed to be ≥80%. It has been reported that mild cognitive impairment (MCI) could already be a significant risk for progression of disabilities in older persons [23], and screening these patients is vital. Similar to frailty, the prevalence of cognitive impairment was significantly different among diagnostic criteria. It is known that MoCA is more sensitive than the MMSE for detecting MCI because it assesses cognitive domain impairment, including executive functioning, attention and concentration, visuospatial skills, and memory. In a report by Trzepacz et al., a score of 25 for MoCA was equivalent to a score of 29 for the MMSE and a score of 26–30 for MoCA was equivalent to a score of 30 for the MMSE [24]. Thus, MoCA may be more sensitive than the MMSE in detecting cognitive impairment. In fact, reports have shown the superiority of MoCA over the MMSE in screening for MCI in patients with DM [25] and heart failure [26].

The prevalence of sarcopenia in our population was considerably higher than that of community-dwelling people in the Asian countries (males, 7.1%; females, 19.8%) [14]. This is natural because most of our subjects were affected by various chronic diseases. Indeed, both DM and heart failure are known to be risk factors for skeletal muscle mass reduction [27, 28]. Similar to our study, Han et al. have reported that the prevalence of sarcopenia in China increased with the accumulation of cardiovascular risk factors, DM, HT, and DL using the AWGS criteria [29]. However, the prevalence of sarcopenia in individuals with these diseases was 11.1–22.2%, which was low compared with that in our subjects [29]; this could be explained by the difference in age of the study subjects as well as the exclusion criteria regarding patients with previous cardiovascular diseases. Notably, among the diagnostic items of sarcopenia, the majority of patients met the criterion of low muscle mass and muscle weakness, whereas almost all did not meet that of walking speed (Table 3). In the AWGS criteria, the cutoff points for SMI and grip strength were slightly lower than those of the European Working Group on Sarcopenia in Older People [30]; however, the cutoff point for walking speed remained unchanged. It is suspected that SMI of the Japanese is considerably smaller than that of the European people reflecting their small body size, whereas in contrast, walking speed in the Japanese older people is comparatively faster [31]. Considering these specific characteristics of Japanese, it might be necessary to produce a more appropriate diagnostic criterion for sarcopenia in the Japanese older persons. In addition to the items needed for the diagnosis of sarcopenia, we performed the one-leg standing and TUG tests. It has been reported that both indices are associated with instrumental ADL status [13, 32] and falls [33, 34].

In this study, we also evaluated the depressive mood, nutritional status, and social support network since several reports have revealed that depressive mood and malnutrition could be risks for frailty [35, 36], and a recent report revealed that the older persons living alone are susceptible to becoming frail [37], which indicates that the lack of social support also could be a crucial risk factor for frailty.

We found that the prevalence of frailty, cognitive impairment, and sarcopenia increased with advancing age; however, the prevalence of sarcopenia plateaued in the subjects > 80 years of age. Few studies have investigated sarcopenia in very old subjects (≥85 years). Our ceiling effect could be accounted for by selection bias. Because our study was held mainly in an outpatient clinic, those who registered and were > 85 years old were relatively healthy and did not represent the general population of the same age. In the Newcastle 85+ study, the authors mentioned that low BMI (≤18.5) was a significant risk factor for the prevalence and incidence of sarcopenia in this age group [38]. In our patients ≥85 years; however, the median BMI was 21.6, and most of the patients had normal nutrition.

It has been reported that DM are associated with high prevalence and incidence of frailty [39, 40], and that HT is also related to prevalent frailty [41, 42], our results stratified by age revealed almost no significant difference in the prevalence of frailty by DM or HT. It is also known that DM is associated with cognitive dysfunction [43] and sarcopenia [44], but no difference was observed except for the prevalence of sarcopenia in the youngest group. This result may also be due to a selection bias in the specialty clinic. Although these subjects became stable, they might have been referred from general practitioners because they had poor control of glucose or blood pressure and multi-morbidities, such as CAD, stroke, or heart failure. These backgrounds of the subjects could have diluted the effects of each single disease, especially in those in the older age groups. For example, it has been reported that chronic heart failure was associated with frailty [45] and cognitive impairment [46]. However, even when considering the bias above, the prevalence of frailty, sarcopenia, and cognitive impairment in the youngest DM group appears to be high, suggesting the importance of taking all possible measures to prevent frailty from occurring at an earlier stage in DM patients.

The prevalence of frailty in the oldest DM group (aged ≥85 years) showed a trend of reduction compared to that in the non-DM group; however, this was not the case for HT, and the reason for this discrepancy remains unclear. Perhaps, some selection or survivorship bias might have influenced these results. Nevertheless, it is unclear why the prevalence of sarcopenia was low in HT subjects aged 80–84 years.

It has been reported in the Japanese population that frailty is associated with both sarcopenia and cognitive decline [47]; however, our results provide valuable new information about how these conditions related to aging overlap with each other. Although the prevalence of cognitive impairment was high, it is noteworthy that almost all of the frail and sarcopenic subjects were cognitively impaired. Alternatively, the prevalence of suspected dementia among the frail and sarcopenic subjects were relatively small. The factors that determine the coincidence of progression of physical function and cognitive decline should further be elucidated by observing this cohort longitudinally.

The strength of our study was that this is the first study to describe the establishment of a frailty clinic with the unique unprecedented backgrounds of our clinic’s patients. Several studies have evaluated frailty status in their own frailty clinics; however, their patients’ backgrounds were quite different from ours. Tavasson et al. reported that the prevalence of frailty in those who visited their original frailty clinic was 54.5%, which was considerably higher than ours [48]. Their registration criterion was “those considered as frail by their physician” so that they could include several patients with functional disabilities; this was evidenced by the low mean gait speed of their participants of 0.78 m/s. The prevalence of frailty in other studies assessed in “geriatric outpatient clinics” were approximately 35% [49, 50], which were close to the prevalence in our study; however, the sample numbers were small (*n* < 200), the studies were conducted in the US or Canada, and although the prevalence of hypertension was higher in one of the studies, there appeared to be few patients with metabolic diseases. Our study appears to be the first that was conducted in Japanese patients with mainly cardiometabolic diseases who were self-supported but at high risk of becoming frail.

Another strength of our study is that we evaluated frailty and cognitive status by using multiple test modalities, including the CHS, CFS, KCL, and TMIG-IC for frailty and the MMSE, HDS-R, MoCA-J, and DASC-21 for cognitive impairment. This study characteristic is also unprecedented. Using these precise datasets, we could determine the index for frailty and cognitive function that is most associated with and most appropriate to predict a specific outcome. The comprehensive assessment using patients with cardiometabolic disease at baseline will help us to explore risk factors for the progression of frailty and cognitive decline in an ongoing 3-year longitudinal prospective observational study concerning frailty in patients with diabetes or heart diseases.

Our study had some limitations. First, the study was conducted with a relatively small sample size to detect a difference in the age- and disease-stratified analyses. Nevertheless, our results have clarified for the first time the prevalence of frailty, sarcopenia, and cognitive impairment in patients with a wide range of age who presented with cardiometabolic diseases. Second, this study was performed only in one Japanese institution. Our results should be confirmed in large multicenter and multiracial studies. In addition, the heterogeneity of our subjects’ backgrounds may make it difficult to apply the results to the general population. We plan to expand the subject samples to include a wide variety of diseases. Third, as this analysis was performed in a cross-sectional study design, the causal associations between cardiometabolic diseases and frailty, sarcopenia, or cognitive impairment remain unknown. To clarify the exact associations, further longitudinal studies are warranted.

## Conclusion

We established a frailty clinic in our institution and selected a cohort to be analyzed in the follow-up studies. The subjects’ statuses of frailty, cognitive function, and sarcopenia were assessed. By using this group of patients, we hope to discover useful information concerning frailty, cognitive impairment, sarcopenia, and other aging-related disabilities in older adults.

## Abbreviations

ADL: Activity of daily living
AWGS: Asian Working Group for Sarcopenia
CAD: Coronary artery disease
CFS: Clinical Frailty Scale
DASC-21: Dementia Assessment Sheet in Community-based Integrated Care System-21 items
DL: Dyslipidemia
DM: Diabetes mellitus
GDS-15-J: Japanese version of the Geriatric Depression Scale 15; HbA1c, glycohemoglobin
HDS-R: Hasegawa’s Dementia Scale for Revised
HT: Hypertension
KCL: Kihon Check List
LSNS-6: Lubben Social Network Scale-6
mCHS: Modified Cardiovascular Health Study
MCI: Mild cognitive impairment
MMSE: Mini-Mental State Examination
MNA-SF: Mini-Nutritional Assessment-Short Form
MoCA-J: Japanese version of the Montreal Cognitive Assessment
SMI: Skeletal Muscle Mass Index
TMIG-IC: Tokyo Metropolitan Institute of Gerontology Index of Competence
TUG: Timed up and go test
